# Thiemann disease and familial digital arthropathy – brachydactyly: two sides of the same coin?

**DOI:** 10.1186/s13023-019-1138-x

**Published:** 2019-06-27

**Authors:** Nadirah Damseh, Jennifer Stimec, Alan O’Brien, Christian Marshall, Ravi Savarirayan, Ali Jawad, Ronald Laxer, Peter Kannu

**Affiliations:** 1 Division of Clinical and Metabolic Genetics, The Hospital for Sick Children and University of Toronto, Toronto, ON M5G 1X8 Canada; 2 Department of Diagnostic Imaging, The Hospital for Sick Children University of Toronto, Toronto, ON Canada; 3 Department of Paediatric Laboratory Medicine and Laboratory Medicine and Pathobiology, The Hospital for Sick Children and University of Toronto, Toronto, ON M5G 1X8 Canada; 4 Division of Rheumatology, Department of Paediatrics and Medicine, The Hospital for Sick Children, University of Toronto, Toronto, ON Canada; 5 Victorian Clinical Genetics Service, Murdoch Childrens Research Institute, The University of Melbourne, Melbourne, Australia; 6 Department of Rheumatology The Royal London Hospital, The University of London, E1 4DG, London, UK; 7 Developmental and Stem Cell Biology, The Hospital for Sick Children, Toronto, ON M5G 1X8 Canada

**Keywords:** TRPV4, Hand, Osteonecrosis, Osteoarthritis, Arthritis

## Abstract

**Background:**

Familial digital arthropathy-brachydactyly (FDAB) and Thiemann disease are non-inflammatory digital arthropathies with many phenotypic similarities. Thirty-three cases of Thiemann disease have been described so far (Mangat et al, Ann Rheum Dis 64:11-2, 2005; Ha et al, Thiemann's disease: a case Report, 2017) but no gene variants have been identified as causative to date. FDAB is reported in only a few patients and has been associated with three heterozygous missense variants in the Transient receptor potential vanilloid 4 (TRPV4) gene. We report a TRPV4 variant in a father and son referred with a diagnosis of Thiemann disease and compare the clinical and radiological features of Thiemann disease with Familial digital arthropathy-brachydactyly (FDAB). We hypothesize that these two entities may be one and the same.

**Methods:**

We describe a father and son referred with a diagnosis of Thiemann disease who were subsequently identified with a heterozygous variant (c.809G > T) in TRPV4. The identical genetic variant was previously reported to cause FDAB. A PUBMED® database search was conducted to retrieve articles related to Thiemann disease and FDAB. We were able to review the clinical and radiological findings of nineteen individuals affected by Thiemann disease and compare them with three families affected by FDAB.

**Results:**

Thiemann disease initially affects the proximal interphalangeal joints and primarily the middle phalangeal bases. In FDAB, the distal phalangeal joints are first affected with the middle phalangeal heads being the primary site of changes. Radial deviation has only been described in FDAB. Our analysis determined that 5 of 20 individuals affected by Thiemann disease have clinical and radiological findings that also fit well with FDAB.

**Conclusion:**

FDAB and Thiemann disease are non-inflammatory digital arthropathies with phenotypic overlap. Although more extensive joint involvement, a distal hand joint preponderance and brachydactyly are expected in FDAB, there are striking clinical and radiological similarities between the two entities. Our analysis suggests that these two phenotypes may represent phenotypic variability of the same entity. Despite many attempts to identify other reported patients affected by Thiemann disease, we were not able to procure DNA from any of the cases to verify our findings. Genetic testing of an affected individual will be crucial in order to provide accurate reproductive genetic counselling about the autosomal dominant nature of this condition.

**Electronic supplementary material:**

The online version of this article (10.1186/s13023-019-1138-x) contains supplementary material, which is available to authorized users.

## Background

Thiemann disease (OMIM 165700) is a rare deforming interphalangeal joint arthropathy of the fingers and toes. First described in 1909, Thiemann reported a 16-year-old male carpenter with painful and progressive proximal interphalangeal (PIP) joint enlargement. There was no family history suggestive of an inherited condition [1]. Subsequently, thirty-two cases of similar hand and foot epiphyseal abnormalities have been reported [2, 3]. Thiemann disease is believed to be autosomal dominant, demonstrating strong penetrance. No causative gene mutation has been identified to date [4, 5]. The classical radiological features described include irregularity, flattening, fragmentation, and broadening of the basal phalangeal epiphyses, followed by joint space narrowing, premature physeal fusion, and phalangeal shortening [6]. The proposed clinical criterion by Handa et al., also include onset before the age of twenty-five, PIP joint swelling and the absence of elevated inflammatory markers [7].

Familial digital arthropathy-brachydactyly (FDAB, OMIM 606835) is an autosomal dominant digital arthropathy first described in 2002 [8]. To date, only two groups have reported affected patients [8–10]. FDAB presents in the first decade as a deforming arthropathy of the interphalangeal, metacarpophalangeal, and metatarsophalangeal joints. There is associated progressive brachydactyly of the middle and distal phalanges of the hands and feet. In 2011, Lamande et al., identified three different heterozygous missense variants in the Transient receptor potential vanilloid 4 (*TRPV4)* gene associated with FDAB. All three variants were shown to reduce TRPV4 channel activity [9, 11].

Here, we describe a father and son referred with a diagnosis of Thiemann disease whom were subsequently identified with a pathogenic *TRPV4* variant. We review the literature on Thiemann disease and FDAB to show the phenotypic overlap. Based on our observations, we suggest that all individuals presenting with a Thiemann like phenotype undergo *TRPV4* mutational analysis to clarify the genetic etiology of their condition.

## Patients and methods

Patient (I) is a 15-year-old male who reported a two-year history of non-painful “crooked” fingers in the absence of trauma. His past medical history was significant for possible Raynaud phenomenon but otherwise noncontributory. On physical examination, his growth parameters were age appropriate. There was radial deviation of the second, third, fourth and fifth terminal phalanges bilaterally. There was asymmetrical involvement of the hands; the third phalanges were most affected and the right-hand digits were more severely affected than the left (Fig. 2, a1). His total hand length was 18 cm (50–75%) and middle finger length was 8 cm (75%). Other joints including those in the feet were normal on examination. His peripheral neurological examination was unremarkable.

Laboratory investigations (white blood cells, erythrocyte sedimentation rate, C-reactive protein, anti-nuclear antibodies, rheumatoid factor, anti-double stranded DNA antibodies, anti-SM antibodies, anti-RNP antibodies, anti-SS-A (RO) antibodies and anti-SS-B (La) antibody) were normal or negative.

Patient (II) is 50 years old and the father of Patient (I). He reported progressive finger joint deformities from the age of 10. He was seen in the Orthopaedic clinic at The Hospital for Sick Children 15 years ago, and a diagnosis of Thiemann disease was considered. He has not required any treatment apart from analgesics for pain. He has also been diagnosed with gout, which occasionally affects his hands and feet. He has mild psoriasis. On examination, he is of above average stature. He had symmetrical hand changes. He had a fixed flexion deformity of the distal interphalangeal (DIP) joints, which were also prominent and radially deviated. His proximal interphalangeal joints were prominent and the range of movement limited. He also had prominent metatarso-phalangeal joints without any limitation to active movement. No other joints were clinically involved. His peripheral neurological examination was unremarkable. There were no deficits in power, sensation or position sense.

An autosomal dominant inheritance was suspected based on the observation of male to male transmission. A Next Generation skeletal dysplasia gene panel (The Hospital for Sick Children, Toronto) identified a heterozygous pathogenic variant (c.809G > T) in the *TRPV4* gene in patient (I) and patient (II). This variant has previously been reported in a patient with familial digital arthropathy-brachydactyly [9].

### Literature review

A PUBMED® database search was conducted in an iterative manner during September–November 2016 to retrieve articles related to Thiemann disease. Search terms included “Thiemann” “Thiemanns” and “Familial digital arthropathy”. A relatively small number of articles exist on the topic. The reference list of each article was reviewed in detail to find additional articles.

Thirty-five articles published between 1954 and 2017 were found, written in different languages (English (*n* = 19), French (*n* = 5), Deutsch (*n* = 4), Polish (n = 1), Romanian (n = 1), Italian (n = 1), undetermined language (*n* = 2)). We excluded documents that were not in English or French. A search using the University of Toronto Libraries online database identified the records of eighteen English and two French articles about Thiemann disease and three English articles about FDAB. These articles were read in full text and the relevant findings were summarized in Table 1.

**Table 1:** Comparison of Thiemann disease and FDAB

	Thiemann disease	Familial Digital Arthropathy and Brachydactyly (FDAB)
Gender	M–F ratio is 1:1.1 (9 males, 10 females, 17 different families)	M–F ratio is 1:1.1 (12 males, 13 females, 4 different families)
Age of onset	8 years −40 years (median age = 24 years)	First decade, earliest reported age was 10 years.
Pattern of inheritance /genetic etiology	Autosomal dominant/ unknown	Autosomal dominant/*TRPV4* variants
Clinical features		
Pain	73% (14/19), usually mild.	Prominent feature
Swelling	75% (15/20)	Prominent feature
Joint movement limitation	73% (14/20)	Present
Symmetrical	85% (17/20)	Prominent feature
Radiological features		
Affected hand joints	95% (19/20): PIPs are more severely affected than DIPs o PIPS 95% (19/20) o DIPs 40% (8/20) o MCPs 20% (4/20)	Constant involvement of PIPs, DIPs and MCPs. (DIPs were more severely affected than PIPs)
Affected feet joints	20% (4/20)- MTPs	By adulthood, all MTPs are affected
Flattening	35% (7/20)	Prominent feature
Irregularities	45% (9/20)	Prominent feature
Short phalanges	25% (5/20)	Prominent feature (progressive)
Broadening	15% (3/20)	Present
Joint space reduction	40% (8/20) o PIPs (8/20) o DIPs (3/20)	Present (in adulthood)
Subchondral cysts	5% (1/20)	Present
Fragmentation	35% (7/20)	Not reported
Lateral deviation	5% (1/20)- ulnar	Prominent feature (Radial>ulnar)

*M* male, *F* female, *PIPs* proximal interphalangeal joints, *DIPs* distal interphalangeal joints, *MCPs* metacarpophalangeal joints, *MTPs* metatarsophalangeal joint

## Results

To date, three families affected by FDAB have been reported and 33 cases of Thiemann disease identified. We were able to review 20 cases of Thiemann disease [see Additional file 1: Table S1]. In 5/20 Thiemann cases, the clinical and radiological descriptions fit well with FDAB ((Miric et al. (1971) (I and II), Ernest et al. (1954), Van der Laan et al. (1986), and Jawdat et al. (2005)) [2, 12–14]. The clinical and radiological features of Thiemann disease and FDAB are summarized in Table 1.

### Clinical findings

The onset of disease in patients with Thiemann disease was in the second decade of life in 65% of patients (13/20), median age was 24 years (range 8 years to 40 years). There were three individuals described with disease onset before the first decade; in two of these individuals, both DIP and PIP joints were affected at the time of initial presentation. Although the condition is described as 'mild' in many of the younger reported patients, this was not a consistent observation. For example, the cases described by Molloy et al. (1978) and Gewanter et al. (1985) presented with a severe joint phenotype at the age of 10 years and 12 years respectively [15, 16].

**Fig. 1:**
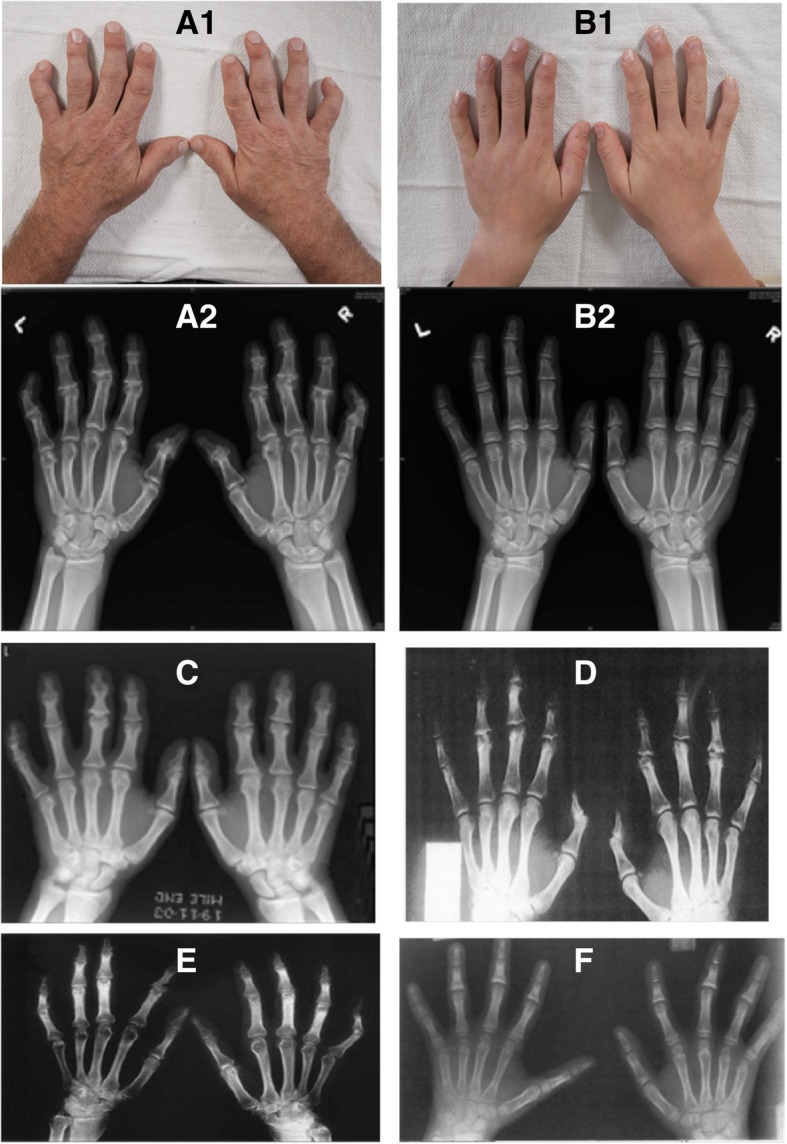
(**a**1, **a**2) patient (II). Multiple bony abnormalities present, particularly involving the heads of the middle phalanges characterized by irregularity, flattening, and radial angulation. Bilateral symmetric marked joint space loss of the 2nd-5th DIP and PIP joints, 1st IP joints, left 5th MCP and right 2nd MCP joints. Osteophyte formation in a similar distribution with a subchondral cyst in the left 3rd DIP. Shortening of the middle phalanges, worst at the second and fifth digit is seen. (**b**1, **b**2) patient (I). Short and broad middle phalanges bilaterally, worst at the 2nd and 5th fingers. Irregularity and sloping of the 2nd -5th middle phalangeal heads with resultant radial deviation. No secondary degenerative changes. (**c**) 19-year-old man reported by Jawad et al. with Thiemann disease. There is flattening and irregularity of the phalangeal epiphyses and broadening of the PIP and DIP joints. There are signs of secondary osteoarthritis including joint space loss and osteophyte formation. Mild shortening of 2nd-5th middle and distal phalanges. (**d**) 17-year-old boy reported by Nurdan Kotevoglu-Senerdem et al. with Thiemann disease. There is irregularity, fragmentation and flattening of the 2nd-4th PIP and DIP joints. (**e**) 25-year-old women reported by Seçkin et al. with Thiemann disease. Note irregularity and flattening of the epiphyses and flexion deformity of both fifth finger PIP joints. There is thickening at the base of all proximal phalanxes and all middle phalanxes were broad. There is narrowing at the third and fourth DIP and fifth PIP joints of both hands. (**f**) Thiemann disease case report of a 10-year-old female by Molloy et al. There are dense sclerotic distal phalangeal epiphyses (ivory epiphyses). There is broadening and irregularity of the 2-5th middle phalangeal bases with premature physeal fusion and relative shortening. Mild ulnar deviation of the right third PIP joint. Note: permissions were obtained from the copyright holder to reuse the images c,d,e and f

Joint involvement was symmetrical in 85% (17/20). Joint pain exacerbated by hand use or cold exposure was reported in 68% (13/20). Soft tissue swelling around the PIP joints, and less commonly the DIP joint, was described in all cases. Other than the hands and feet, no individuals reported any other large joint involvement or features of a systemic illness. While radial deviation of the interphalangeal joints was common in the FDAB cases, this was not reported in Thiemann disease. Few patients affected by Thiemann disease developed ulnar deviation of the hand joints (Miric et al. (1971), Molloy et al. (1978) see Fig. 1-f, Kotevoglu-Senerdem et al. (2003) see Fig. 1-d) [12, 15, 17].

### Radiological findings

Common radiological findings reported in Thiemann disease include proximal and distal joint irregularity, subchondral cysts, joint space reduction, erosions, flattening of the distal phalangeal base, and a slight reduction in the middle and distal phalangeal lengths. However, these features were also noted in FDAB (Fig. 1). Thiemann disease initially affects the proximal interphalangeal joints and primarily the middle phalangeal bases, unlike FDAB where distal phalangeal joints are first affected with the heads of the middle phalanges the primary site of change.

Almost always, both interphalangeal joints of the hand (PIP > DIP) were affected in Thiemann disease while the feet were affected in 20% (4/20) of individuals. Shortening of the hand phalanges was documented in 25% (5/20) of individuals. The thumb was typically spared. Shortening and broadening of the phalangeal and metacarpal bones was found in 30% (6/20) of individuals. In three individuals, carpal bone abnormalities were described. In the feet, the metatarsophalangeal joints were typically affected but interphalangeal joint involvement less common.

## Discussion

Thiemann disease represents a progressive hand and foot arthropathy with variable expressivity and an autosomal dominant pattern of inheritance [1]. Curiously, a few mildly affected individuals who made a complete recovery with no lasting signs of arthritis have been described [14, 15]. Thiemann disease may present as early as age 4 years but is more commonly diagnosed in the early teen years [6]. In a typical case, an affected individual reports relatively painless swelling of the proximal interphalangeal joints or an inability to use the digit [3, 6, 11, 14]. Proximal interphalangeal joint involvement, and to a lesser extent, involvement of the first metatarsophalangeal and metacarpophalangeal joints is well described [1, 3, 10–13]. Mild shortening of the phalanges is also commonly reported [4]. On the other hand, FDAB is a more aggressive arthropathy developing in the first decade of life. Unlike Thiemann disease, the interphalangeal, metacarpophalangeal and metatarsophalangeal joints are all typically affected and joint pain is a significant feature. Similar to Thiemann disease, the thumb is frequently spared and the hands more involved than the feet. In both entities, the remainder of the skeleton is clinically and radiographically unaffected [9]. We found the formulated radiological criteria suggested by Melo-Gomes et al. and the revised tentative radiological criteria by Van der Laan et al. for Thiemann disease no more sensitive in eliminating the possibility of FDAB.

The underlying pathological processes in Thiemann disease and FDAB are believed to be different. Thiemann disease is classified as a juvenile osteochondritis similar to Legg–Calvé–Perthes disease and Scheuermann disease [12]. Pathological analysis is only available from a single case where the finger joint showed varying degrees of epiphyseal cartilage necrosis without an inflammatory response [5]. FDAB is hypothesized to result from an arrest of bone growth or bone resorption at the joint subchondral region. Since the earliest observed changes are the deformed and flattened proximal articular surfaces with intact distal articular surfaces and joint spaces, Amor et al. 2011 hypothesized that brachydactyly is secondary to the joint disease in FDAB [8]. We note however, the *TRPV4* mutation described in this report, which has previously been described to cause FDAB, was not associated with brachydactyly. We also believe the epiphyseal changes affecting the middle phalanges of patient (II) could represent avascular necrosis of the radial aspect of the phalangeal condyles. Upon collapse of the condyles, the distal interphalangeal joints would manifest a radial deviation.

Based on the data presented here, we believe that localization of the initial most affected joint helps in discriminating between the two entities. In Thiemann disease, the proximal interphalangeal joints are first affected. FDAB starts in the most distal hand joints, progressively involving the proximal interphalangeal joints and eventually, the metaphalangeal and metatarsophalangeal joints. As the disease progresses, the distal interphalangeal joints develop radiological deformities and restricted movement compared to the proximal interphalangeal joints. Radial deviation of the phalanges is documented in FDAB, but has not been reported in Thiemann disease [12]. More joints are affected in FDAB and pain seems to be a more striking component of the phenotype.

**Fig. 2:**
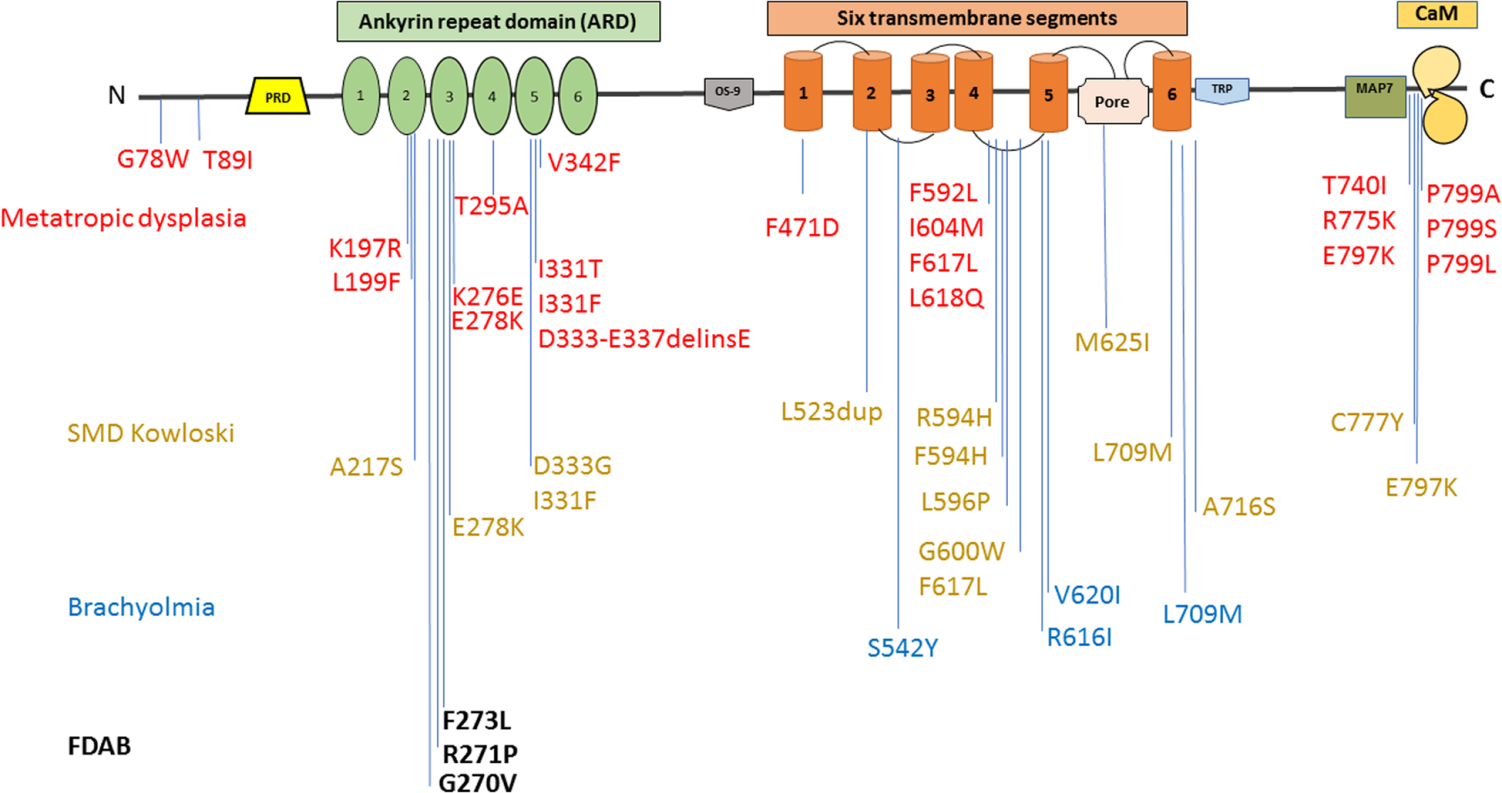
Protein sequence and disease-causing mutations in TRPV4. PRD, protein rich domain. CaM, calmodulin. MAP7, microtubule-associated protein 7

Familial Thiemann disease shows an equal sex distribution, while sporadic cases demonstrate a two-third’s male predominance [6]. No specific radiological differences exist between these two groups of patients and the genetic cause of Thiemann disease is thus far unknown. In the family we describe, a *TRPV4* variant previously reported to cause FDAB was identified. To date, only three different *TRPV4* variant causing FDAB has been reported. TRPV4 forms a Ca2 + −permeable cation channel that is stimulated by heat and mechanical stress. All reported FDAB mutations to date are fully penetrant and affect the third finger of the TRPV4 intracellular ankyrin-repeat domain resulting in a reduction of channel activity and impairment of cartilage hemostasis [9, 11]. Gain-of-function *TRPV4* variants are associated with other phenotypes varying from severe skeletal dysplasias to peripheral neuropathies (Fig. 2). Recently, a novel gain of function *TRPV4* variant was associated with inherited osteonecrosis of the femoral head [18].

## Conclusion

FDAB and Thiemann disease are non-inflammatory digital arthropathies presenting in the first two decades of life with many phenotypic similarities. Neither condition seems to affect other joints. *TRPV4* variants cause FDAB while the familial nature of Thiemann disease is consistent with a genetic etiology. Our report describing a *TRPV4* variant in a father and son referred with a diagnosis of Thiemann disease suggests the historical accounts of these two different phenotypes may be inaccurate in some instances, and that they may be different manifestations of the same disease. Although more extensive joint involvement, a distal hand joint preponderance and brachydactyly are expected in FDAB, there are striking radiological similarities between the two entities. Despite many attempts to identify other affected patients, we were unfortunately unable to procure DNA from any of the previously reported cases to verify our findings. Genetic testing of an affected individual will be crucial in order to provide accurate reproductive genetic counselling about the autosomal dominant nature of this condition.

## Additional file

Additional file 1: **Table S1.** Clinical and radiological manifestations of Thiemann’s disease in all reviewed cases. (DOCX 54 kb)

## Data Availability

All articles included in the review are listed in the references. Articles were identified in Pubmed database and access to full text is dependent on journal and institutional constraints.

## Abbreviations

DIP: Distal interphalangeal
FDAB: Familial digital arthropathy-brachydactyly
PIP: Proximal interphalangeal
TRPV4: Transient receptor potential vanilloid 4
