# Supplementary material for: Thiemann disease and familial digital arthropathy – brachydactyly: two sides of the same coin?
Source: Orphanet J Rare Dis. 2019 Jun 27;14:156. doi: 10.1186/s13023-019-1138-x (PMC6598251; doi:10.1186/s13023-019-1138-x)
Supplement: Supplementary file 1 — Table S1. Clinical and radiological manifestations of Thiemann’s disease in all reviewed cases. (DOCX 54 kb) [file 13023_2019_1138_MOESM1_ESM.docx]

|  | **Case 1**  Ernest et al 1954(1) | **Case 2**  Iversen 1955(2) | **Case 3**  Allison et al 1958 (I)(3) | **Case 4**  Allison et al 1958 (II)(3) | **Case 5**  Miric et al., 1971 (I)(4) | **Case 6**  Miric 1971 (II)(4) |
| --- | --- | --- | --- | --- | --- | --- |
| **Sex** | Male | Male | Female | Female | Male | Male |
| **Age** | 20 yrs | 13 yrs | 49 yrs | 61 yrs | 17 yrs | 50 yrs |
| **Onset** | 15 yrs | 13 yrs | NA | 12 yrs | 16 yrs | NA |
| **Presentation** | Swelling (Rt 3^rd^ finger) | Swelling (Rt 3^rd^ finger PIP) | unrelated complaints | Deformed & enlarged hand joints. | Deformation of the fingers | Deformation of the fingers |
| **Bilateral** | + | - | + | + | + | + |
| **Hand joints** | PIPs and DIPs. Short fingers. | Rt 3^rd^ PIP. Some limitation of full flexion. | All PIPs and DIPs bi, with movement limitation | All PIPs and DIPs. Short terminal phalanges. | 2^nd^ & 3^rd^ PIPs bi. Mild flexion at all DIPs. Ulnar inflexion of 3^rd^ PIPs. | Bi deformation of 2^nd^-5^th^ PIPs and DIPs. |
| **Feet joints** | - | - | Hallux valgus | - | NA | NA |
| **Other joints** | Knee joint swelling & discomfort | - | - | - | NA | NA |
| **Pain/trigger** | Mild pain | Mild pain | - | - | Pain with hands joints flexion. | Pain with flexion on hands joints. |
| **erythema** | - | - | - | - | - | - |
| **swelling** | + | + | + | + | + | + |
| **Deviation** | - | - | - | - | ulnar | - |
| **Family history** | Positive | - | Positive | Positive | Positive | Positive |
| **Systemic symptoms** | - | - | - | - | - | - |
| **Inflammatory markers** | Normal | NA | Normal | Normal | Normal | Normal |
| **Radiography** | Short/broad metacarpals and phalanges | Rt. Middle phalynx epiphysis sequestration. | Small phalanges. Lipping and narrowing of the joint space of interphalangeal joints with some flattening as osteoarthritis. Slight changes of carpal bones. | Small phalanges. Narrowing of the joint space of interphalangeal joints with some flattening. mild changes of carpal bones. changes of MCPs of all digits. | Irregularities (hands 3^rd^ PIPs, 3^rd^ - 5^th^ DIPs). Flattened and fragminted Rt 3^rd^ MC head. bi fragmentation of the 5^th^ MT base, flattening and irregularity of the of Rt 1^st^ MTJ head. apophysitis calcaneum. | Bi irregularities of 2^nd^-5^th^ PIPs and DIPs. Abnormal 2^nd^,4^th^ , 5^th^ MC heads. Rt 5^th^ MT apophysis fragmentation. deformed distal phalanges of first toes. |

**Table 1:** Clinical and radiological manifestations of Thiemann’s disease in all reviewed cases

|  | **Case 7**  Cullen 1970(5) | **Case 8**  Rubinstein 1975(6) | **Case 9**  Molloy 1978(7) | **Case 10**  Melo-gomes 1981(8) | **Case 11**  Gewanter 1985 (I)(9) | **Case 12**  Gewanter 1985 (II)(9) | **Case 13**  Van der Laan 1986(10) |
| --- | --- | --- | --- | --- | --- | --- | --- |
| **Sex** | Male | Male | Female | Female | Male | Female | Female |
| **Age** | 17 yrs | 54 yrs | 10 yrs | 15 yrs | 13 yrs | 12 yrs | 9 yrs |
| **Onset** | 16 yrs | 40 yrs | 10 yrs | 12 yrs | 13 yrs | 8 yrs | 8 yrs |
| **Presentation** | Pain, swelling and stiffness of the Rt 3^rd^ finger | unrelated complaints | Pain & swelling of all PIPs | Swelling of the PIP of the 3^rd^ finger bi | Swelling of both hands Rt 2^nd^ PIPs | Bi 2^nd^ & 5^th^ fingers pain & swelling. short fingers & toes | Mild pain in lt 3^rd^ finger PIP |
| **Bilateral** | + | + | + | + | + | + | - |
| **Hand joints** | Rt 3^rd^ PIP swelling with limited flexion. | Thickening & contracture of all PIPs. Mild DIPs flexion limitation. | Swelling and deformity of all PIPs bi with extreme tenderness. | Painless Swelling of the PIP of the 3^rd^ finger bi. | swelling & tenderness of Rt 2^nd^ & 5^th^ PIPs / minimal loss of flexion. | 2^nd^ & 5^th^ short middle phalanges. Bi PIPs swelling. | Mild movement limitation of Rt 3^rd^ DIP. |
| **Feet joints** | NA | Bi hallux valgus | - | - | - | Bi Short 2^nd^ - 5^th^ phalanges. | NA |
| **Other joints** | NA | - | - | - | - | - | - |
| **Pain** | + | Mild pain | + | Mild /cold exposure. | + | Pain with piano playing & writing | Mild pain |
| **erythema** | - | - | - | - | - | - | - |
| **swelling** | + | + | + | + | + | + | - |
| **Deviation** | - | - | - | - | - | - | - |
| **Family**  **history** | Positive | Positive | - | - | - | - | Positive |
| **Systemic symptoms** | - | - | - | - | - | - | - |
| **Inflammatory markers** | Normal | Normal | Normal | Normal | Low IgA | Normal | NA |
| **Radiography** | Narrowing, fragmentation and sclerosis of the 3^rd^ middle phalanx epiphysis. Breaking fingers basal epiphyses. | Narrowed PIP joints, mainly 2^nd^ - 3^rd^ bony overgrowth and shortening of middle phalanges. 1^st^ MTP joint osteoarthritic changes. | Extreme epiphyseal abnormalities; destruction thinning of the epiphyses,  expansion of the base of the middle phalanges | Flattening & narrowing of 3^rd^ PIPs bi. Rt 3^rd^ PIP fragmentation. Widening of 3^rd^ & 4^th^ middle phalange bases bi. 2 small cystic lesions at Rt 3^rd^ middle phalange. | Rt 2^nd^ & 5^th^ PIPs changes. Irregularities of both 5^th^ digits distal middle phalanges & con shaped epiphysis. radiolucencies of 2^nd^ toes. | Bi symmetrical shortened 2nd and 5th digits. Bi 2nd - 5th toes Shortened phalanges with fused epiphyses. | Ivory epiphysis. Sclerotic /irregular of 3rd fingers middle phalanx epiphysis. |

|  | **Case 14**  Gonzalez Dominguez et al., 1991(11) | **Case 15**  Handa 1998(12) | **Case 16**  Seçkin 1999(13) | **Case 17**  Kotevoglu-Senerdem 2003 (I)(14) | **Case 18**  Kotevoglu-Senerdem 2003 (II)(14) | **Case 19**  Jawdat 2005(15) | **Case 20**  Kim 2017(16) |
| --- | --- | --- | --- | --- | --- | --- | --- |
| **Sex** | Female | Male | Female | Male | Male | Male | Male |
| **Age** | 11 yrs | 15 yrs | 25 yrs | 17 yrs | 14 yrs | 19 yr | 10 yrs |
| **Onset** | NA | 12 yrs | 16 yrs | 14 years | 13 yrs | 14 yrs | 10 yrs |
| **Presentation** | Deformation and swelling of all PIPs | swelling of PIP of both hands | Pain, swelling & stiffness of Rt 5^th^ PIP | Swelling of the hands 3^rd^ PIPs bi | Swelling of both hands 3^rd^ PIPs. | Swelling of hands DIPs & PIPs bi. | Swelling & mild pain in Rt 5^th^ finger |
| **Bilateral** | + | + | + | + | + | + | - |
| **Hand joints** | All PIPs | fixed flexion deformity of fifth fingers. | All PIPs enlargement. Decrease movement at 3^rd^ and 5^th^ PIPs bi. | Swelling and stiffness of 2^nd^- 4^th^ PIPs bi. | Swelling of 3^rd^ PIPs bi. | All PIPs & DIPs are swollen with limited flexion. | fusiform swelling in PIP of Rt 5^th^  finger |
| **Feet joints** | NA | - | - | - | - | NA | - |
| **Other joints** | Dorsal scoliosis | - | - | NA | NA | NA | - |
| **Pain** | - | - | mild/ activity, cold exposure | - | NA | +/heavy activities | Mild pain |
| **erythema** | - | - | - | NA | NA | - | - |
| **swelling** | + | + | + | + | + | + | + |
| **Deviation** | - | - | - | NA | NA | - | - |
| **Family**  **history** | NA. | - | NA | Positive | Positive | Positive | - |
| **Systemic symptoms** | - | - | - | NA | NA | - | - |
| **Inflammatory markers** | Normal | Normal | Normal | Normal | Normal | Normal | Normal |
| **Radiography** | Short lt 2^nd^ MCP, irregularity of the epiphyses of the middle phalanges. | Irregularity of 2^nd^ phalanx (proximal) & epiphyses of 4^th^ & 5^th^ fingers bi. PIP joints fusion. | Irregular & flat epiphyses. Flexion deformity of 5^th^ PIPs.  Thickening at bases of all proximal & middle phalanxes.  narrowing of 3^rd^ & 4^th^ DIP & 5^th^ PIP bi. | Irregularity, fragmentation, and flattening of 2^nd^-4^th^ PIPs, 2^nd^-4^th^ DIPs.  irregularity and cupping  at the bases of the phalanges.  slight change at the Rt 1^st^ MTP joint. | The joint spaces were  normal, the epiphyses were not closed, and  there was not frank fragmentation. The feet  were also normal. | flattening, broadening, fragmentation,  irregular opacification of the phalangeal epiphyses | Normal |

**References**

**2**

**2**

1. Shaw EW. Avascular necrosis of the phalanges of the hands (Thiemann's disease). Journal of the American Medical Association. 1954;156(7):711-3.

2. Iversen J. Aseptic necrosis of phalangeal epiphysis (Thiemann's disease). Acta chirurgica Scandinavica. 1956;110(6):494-7.

3. Allison AC, Blumberg BS. Familial osteoarthropathy of the fingers. The Journal of bone and joint surgery British volume. 1958;40-b(3):538-45.

4. Miric V, Jankulov D, Tomasevic M. [2 cases of Thiemann's disease]. Revue du rhumatisme et des maladies osteo-articulaires. 1971;28(10):653-4.

5. Cullen JC. Thiemann's disease. Osteochondrosis juvenilis of the basal epiphyses of the phalanges of the hand. Report of two cases. The Journal of bone and joint surgery British volume. 1970;52(3):532-4.

6. Rubinstein HM. Thiemann's disease. A brief reminder. Arthritis and rheumatism. 1975;18(4):357-60.

7. Molloy MG, Hamilton EB. Thiemann's disease. Rheumatology and rehabilitation. 1978;17(3):179-80.

8. Melo-Gomes JA, Melo-Gomes E, Viana-Queiros M. Thiemann's disease. The Journal of rheumatology. 1981;8(3):462-7.

9. Gewanter H, Baum J. Thiemann's disease. The Journal of rheumatology. 1985;12(1):150-3.

10. van der Laan JG, Thijn CJ. Ivory and dense epiphyses of the hand: Thiemann disease in three sisters. Skeletal radiology. 1986;15(2):117-22.

11. Gonzalez Dominguez J, Collantes Estevez E, Alcalde Perez A, Beltra de Hoyos M, Cuadrado Lozano MJ, Martinez Sanchez FG. [Thiemann's disease. Apropos of a new case]. Revue du rhumatisme et des maladies osteo-articulaires. 1991;58(3):201-2.

12. Handa R, Aggarwal P, Wali JP. A young boy with deforming arthropathy. Annals of the rheumatic diseases. 1998;57(2):79-80.

13. Seckin U, Ozoran K, Polat N, Ucan H, Tutkak H. Thiemann's disease: a case report. Rheumatology international. 1999;18(4):157-8.

14. Kotevoglu-Senerdem N, Toygar B, Toygar B. Thiemann disease. Journal of clinical rheumatology : practical reports on rheumatic & musculoskeletal diseases. 2003;9(6):359-61.

15. Mangat P, Jawad AS. Case number 32: Thiemann's disease. Annals of the rheumatic diseases. 2005;64(1):11-2.

16. Horta-Baas G, Vergara-Sanchez I, Romero-Figueroa MDS. Familial digital arthropathy-brachydactyly: An infrequent cause of joint deformity in adolescents. Medicina clinica. 2017;149(11):512-3.
